# Macroscopic Endoscopy Findings in Microscopic Colitis

**DOI:** 10.1016/j.gastha.2026.101084

**Published:** 2026-08-11

**Authors:** Daniel B. Amusin, Ryan A. Nock, Iris Chiou, Andrew Moore, Kyle Geary, Michael Sprang, Omar Khan, Eugene F. Yen

**Affiliations:** 1 Medical Scientist Training Program, Northwestern University Feinberg School of Medicine, Chicago, Illinois; 2 Division of Gastroenterology, NorthShore University Health System, Evanston, Illinois; 3 Division of Gastroenterology and Hepatology, Northwestern University Feinberg School of Medicine, Chicago, Illinois

**Keywords:** Collagenous Colitis, Lymphocytic Colitis, Endoscopy

## Abstract

**Background and Aims:**

Microscopic colitis (MC) is a condition characterized by abnormal histology in the absence of endoscopic findings. However, case reports of endoscopic findings in MC have been published. We compared the prevalence of such findings in patients with MC and controls being evaluated for chronic diarrhea, as well as whether endoscopic findings are associated with MC severity.

**Methods:**

Endoscopy images from patients in our institutional MC registry and controls presenting for colonoscopy for evaluation of chronic diarrhea without MC were reviewed by a panel of physicians for the presence of endoscopic findings: abnormal vascularity, mucosal tears, scarring, nodularity, and scalloping. In patients with MC, degree of clinical severity was compared between patients with and without endoscopic findings.

**Results:**

In our cohort of 275 patients, endoscopic findings were present in 41 of 168 (24.4%) of patients diagnosed with MC and 11 of 107 (10.3%) of those without MC (*P*-adj = .03). Scalloping (*P*-adj = .04) and abnormal vascularity (*P*-adj < .01) were associated with the diagnosis of MC; however, when cohorts were matched by risk factors for MC, this relationship was attenuated. There were no significant differences between endoscopic findings and MC subtypes (lymphocytic/collagenous colitis). Finally, no significant relationship was found between the presence of endoscopic findings and MC disease severity.

**Conclusion:**

Our results suggest that subtle endoscopic findings are more common in patients with MC than those with other causes of chronic diarrhea. While findings are not unique to MC, clinicians should be aware of macroscopic findings seen in patients with MC.

## Introduction

Microscopic colitis (MC) is a chronic inflammatory condition that is traditionally diagnosed by histology in the presence of endoscopically normal colonic mucosa. However, there have been reports describing abnormal endoscopic appearance in some cases of MC. These findings include abnormal vascular patterns, nodularity of mucosa, lacerations, and a mosaic pattern/scalloping of mucosal folds, similar to findings in celiac disease.^1^, ^2^, ^3^, ^4^, ^5^, ^6^, ^7^, ^8^, ^9^, ^10^, ^11^, ^12^, ^13^

Despite recognition of endoscopic abnormalities in the setting of MC, the prevalence of these findings and their role in assisting with diagnosis in a group undergoing colonoscopy with biopsy to evaluate chronic diarrhea is unknown. Lack of control subjects and studies of limited sample size present insufficient evidence of an association between MC and such findings. Furthermore, the effect of endoscopic findings on clinical symptoms in patients with MC remains unexplored. Thus, the aim of this study was to investigate the prevalence of endoscopic abnormalities in patients with MC compared to diarrhea controls and whether endoscopic findings in patients with MC associate with clinical severity.

## Methods

### Patient Selection

Two groups of patients were retrospectively identified in this cross-sectional study. Cases of MC were identified from our institution’s prospectively maintained MC registry from 2009 to 2020. Patients were eligible for enrollment in the registry following a diagnosis of MC based on colonoscopy evaluation of chronic watery diarrhea. MC, and subtypes lymphocytic colitis (LC) and collagenous colitis (CC), were solely defined by histologic criteria used in randomized controlled trials for MC and confirmed by an expert gastrointestinal pathologist. CC was defined by thickness of the collagenous subepithelial table >10 μm, inflammation in the lamina propria consisting of mainly lymphocytes and plasma cells, lack of crypt architectural distortion, and regenerative-appearing changes in the surface and/or crypt epithelium. LC was defined by intraepithelial lymphocytes >20 per 100 epithelial cells in the subjective area of highest lymphocyte density, inflammation primarily composed of lymphocytes and plasma cells, and regenerative-appearing changes in the surface and/or crypt epithelium.^14^^,^^15^ All patients enrolled in the registry for which endoscopy images of their index colonoscopy were available were included in the study. Control patients were those who had undergone outpatient colonoscopy investigation of chronic watery diarrhea with normal biopsies during a study evaluating the frequency of medication use among patients with and without MC.^16^ For both the MC and the control population, random biopsies were taken in a similar fashion from both the right and left colon. Left colon biopsies included the descending and sigmoid colon, but not the rectum. Patients were excluded if they had typical overt findings and histology consistent with a separate diagnosis (ie, Crohn’s disease, ulcerative colitis, malignancy).

Clinical characteristics were collected from patients in both groups. For patients enrolled in the MC registry, demographics, symptoms, smoking and alcohol status, and medication use at the time of their endoscopy were collected either at the time of diagnosis or at a follow-up appointment within 3 months of the index colonoscopy. Severe MC was defined as greater than 6 bowel movements per day, incontinence, or nocturnal bowel movements. Control patients were surveyed on clinical symptoms and medication use prior to their colonoscopy. The primary study outcome was overt colonoscopy findings in patients with MC compared to those without MC.

### Endoscopy Image Review

**Figure 1:**
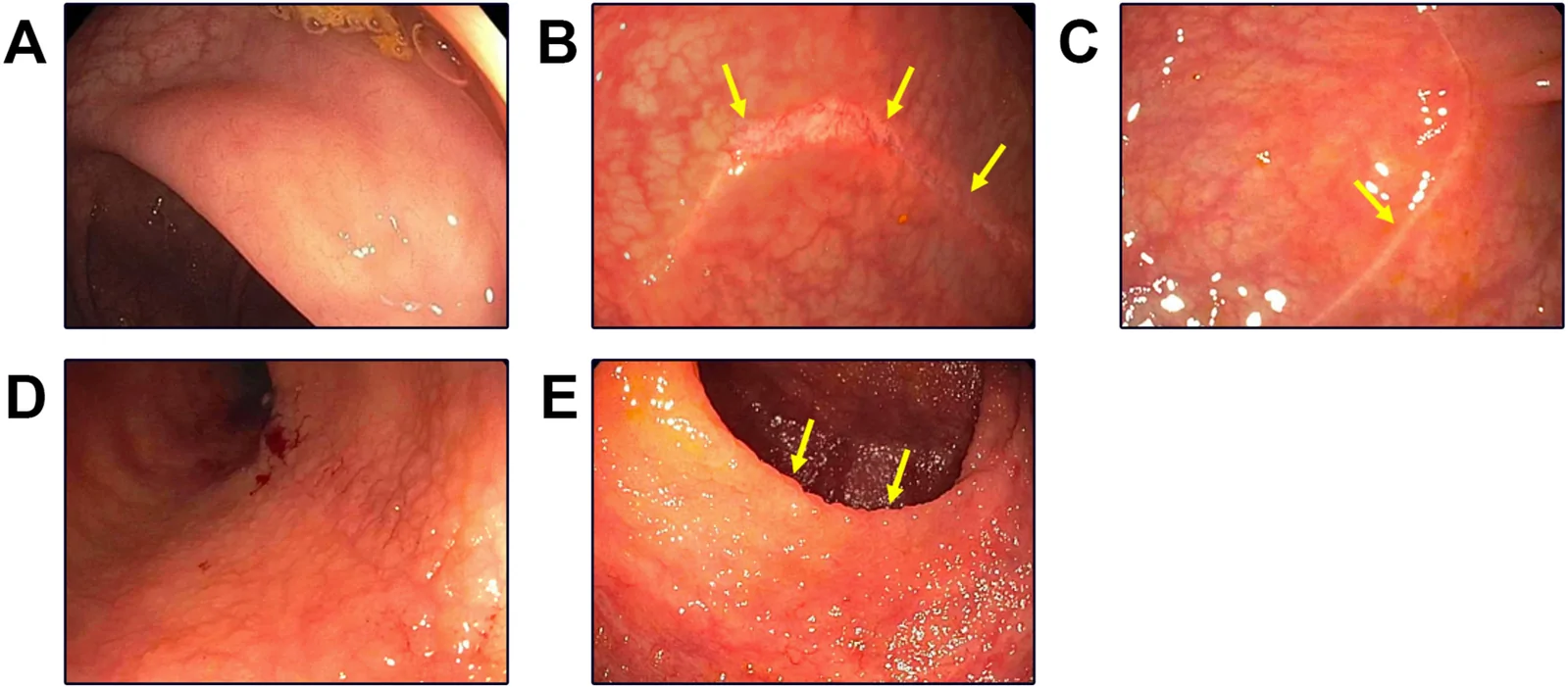
Endoscopic findings in microscopic colitis. The panels in this figure demonstrate endoscopic abnormalities evaluated in this study: (A) Abnormal vascularity. (B) Mucosal tear. (C) Scarring. (D) Nodularity. (E) Scalloping.

Three expert gastroenterologists (E.Y., M.S., O.K.) and 3 gastroenterology fellows (R.N., K.G., A.M.) were trained to detect endoscopic abnormalities using a library of images from previously published case reports.^1^, ^2^, ^3^, ^4^ Next, high-definition white light endoscopy images reflecting the cecum, ascending, transverse, descending, sigmoid colon, rectum and, in most cases, the terminal ileum (median 8 per patient) were obtained from the medical records of patients with and without MC. For patients with MC, only images from the index colonoscopy were reviewed. Therefore, all images for both MC and control patients reflected only their diagnostic colonoscopy for evaluation of chronic watery diarrhea. Images were deidentified and displayed in random order to each of the blinded reviewers for independent evaluation of the presence of vascular abnormalities (Figure 1A), mucosal breaks (longitudinal lacerations and cat scratch pattern; Figure 1B), scar/cicatricial pattern (Figure 1C), and surface alterations (nodularity/mosaic pattern; Figure 1D) or scalloping of mucosal folds (Figure 1E). Vascular abnormalities were loosely defined intentionally to include loss of vascularity, vascular pruning, or hypervascularity. Any images on which reviewers disagreed were displayed for the panel as a group, and a consensus was reached.

### Statistical Analysis

Demographic differences among cohorts and the relationship between endoscopic findings and MC were evaluated using Fisher’s exact and Welch’s *t*-test. To control for known risk factors of MC, a subset of patients with and without MC were matched by age, gender, smoking status, and non-steroidal anti-inflammatory drug use via the nearest-neighbor propensity score method.^17^ Furthermore, in patients with MC, the relationship between endoscopic findings and symptom severity at the time of colonoscopy was evaluated using Fisher’s exact tests. Any missing data were excluded from respective analyses. Statistical significance was set at *P* < .05. To control for the family-wise error rate when testing the relationship between endoscopic findings and MC or markers of MC severity, Bonferroni’s correction was applied to calculate adjusted *P* values (*P*-adj). All statistical analysis was performed using R version 4.5.0.^18^

### Ethical Considerations

This study was approved by the NorthShore University HealthSystem Research Institute Institutional Review Board.

## Results

### Endoscopic Findings and Microscopic Colitis

In our cohort of 275 patients with chronic diarrhea evaluated by colonoscopy with biopsies, 168 had a diagnosis of MC (61.1%), of which 110 had LC (65.5%) and 58 CC (34.5%), and 107 patients were negative for MC (38.9%). Patients diagnosed with MC were more likely to be female and older (*P* ≤ 0.01). Endoscopic findings among patients with and without MC are displayed in Table 1. Findings were identified in a total of 52 patients (18.9%) and were more common among patients with MC (24.4%) than those without MC (10.3%, *P*-adj = .03). Among individual classes of findings, only abnormal vascularity (8.9% vs 0.0%, *P*-adj < .01) and scalloping (8.9% vs 0.9%, *P*-adj = .04) were significantly more common among patients with MC than those not diagnosed with MC. No significant differences in endoscopic findings were seen between patients with MC subtypes of CC and LC.

**Table 1:** Demographics and Endoscopic Findings

	Full cohort (%), N = 275	Lymphocytic colitis (%), N = 110	Collagenous colitis (%), N = 58	All MC (%), N = 168	No MC (%), N = 107	*P* value (all MC vs No MC)	*P*-adj (all MC vs no MC)
Female	204 (74.18)	84 (76.36)	50 (86.21)	134 (79.76)	70 (65.42)	.01	-
Age, mean ± SD	59.49 ± 14.81	61.86 ± 15.79	63.09 ± 10.57	62.29 ± 14.19	55.11 ± 14.77	<.01	-
Smoking^a^							
Current	29 (10.58)	14 (12.73)	7 (12.07)	21 (12.50)	8 (7.55)	.07	-
Former	98 (35.77)	45 (40.91)	21 (36.21)	66 (39.29)	32 (30.19)		
Never	147 (53.65)	51 (46.36)	30 (51.72)	81 (48.21)	66 (62.26)		
NSAID use^b^	96 (42.48)	37 (45.68)	22 (57.89)	59 (49.58)	37 (34.58)	.03	-
Endoscopic findings							
Abnormal vascularity	15 (5.45)	12 (10.91)	3 (5.17)	15 (8.93)	0 (0.0)	<.01	<.01
Mucosal tears	3 (1.09)	1 (0.91)	2 (3.45)	3 (1.79)	0 (0.0)	.28	1.00
Scalloping	16 (5.82)	8 (7.27)	7 (12.07)	15 (8.93)	1 (0.93)	.01	.04
Scarring	8 (2.91)	2 (1.82)	1 (1.72)	3 (1.79)	5 (4.67)	.27	1.00
Nodularity	26 (9.45)	11 (10.00)	9 (15.52)	20 (11.90)	6 (5.61)	.09	.56
Any	52 (18.91)	26 (23.64)	15 (25.86)	41 (24.40)	11 (10.28)	<.01	.03

NSAID, non-steroidal anti-inflammatory drug.

This Table displays demographic information among cohorts and the frequency at which endoscopic findings were found in all patients, those without microscopic colitis (MC), those with MC, and in the subtypes of collagenous colitis (CC) and lymphocytic colitis (LC). Data on use of non-steroidal anti-inflammatory medications (NSAIDs) were available in 226 patients (119 MC, 107 No MC).

a Smoking status was unavailable for 1 patient who did not have MC.

b NSAID use status was unavailable for 29 patients with LC and 20 patients with CC.

To control for significant differences among known risk factors for MC between groups, a subset of patients with and without MC was matched in a one-to-one ratio by age, gender, smoking status, and non-steroidal anti-inflammatory drug use. This yielded 2 groups of 79 patients. The presence of abnormal vascularity varied between groups (7.59% with MC, 0% no MC, *P*-adj = .17) but was not statistically significant (Table 2).

**Table 2:** Endoscopic Findings Controlled for Risk Factors of MC

	MC (%)	No MC (%)	*P* value	*P*-adj
Abnormal vascularity	6 (7.59)	0 (0.00)	.03	.17
Mucosal tears	1 (1.27)	0 (0.00)	1.00	1.00
Scalloping	2 (2.53)	1 (1.27)	1.00	1.00
Scarring	2 (2.53)	4 (5.06)	.68	1.00
Nodularity	5 (6.33)	6 (7.59)	1.00	1.00
Any finding	13 (16.46)	10 (12.66)	.65	1.00

A subset of patients with and without microscopic colitis (MC, N = 79 per group) were matched for known risk factors of microscopic colitis (MC): gender, age, smoking status, and non-steroidal anti-inflammatory used. Presence of macroscopic findings was compared between these groups.

### Endoscopic Findings and Microscopic Colitis Disease Severity

**Figure 2:**
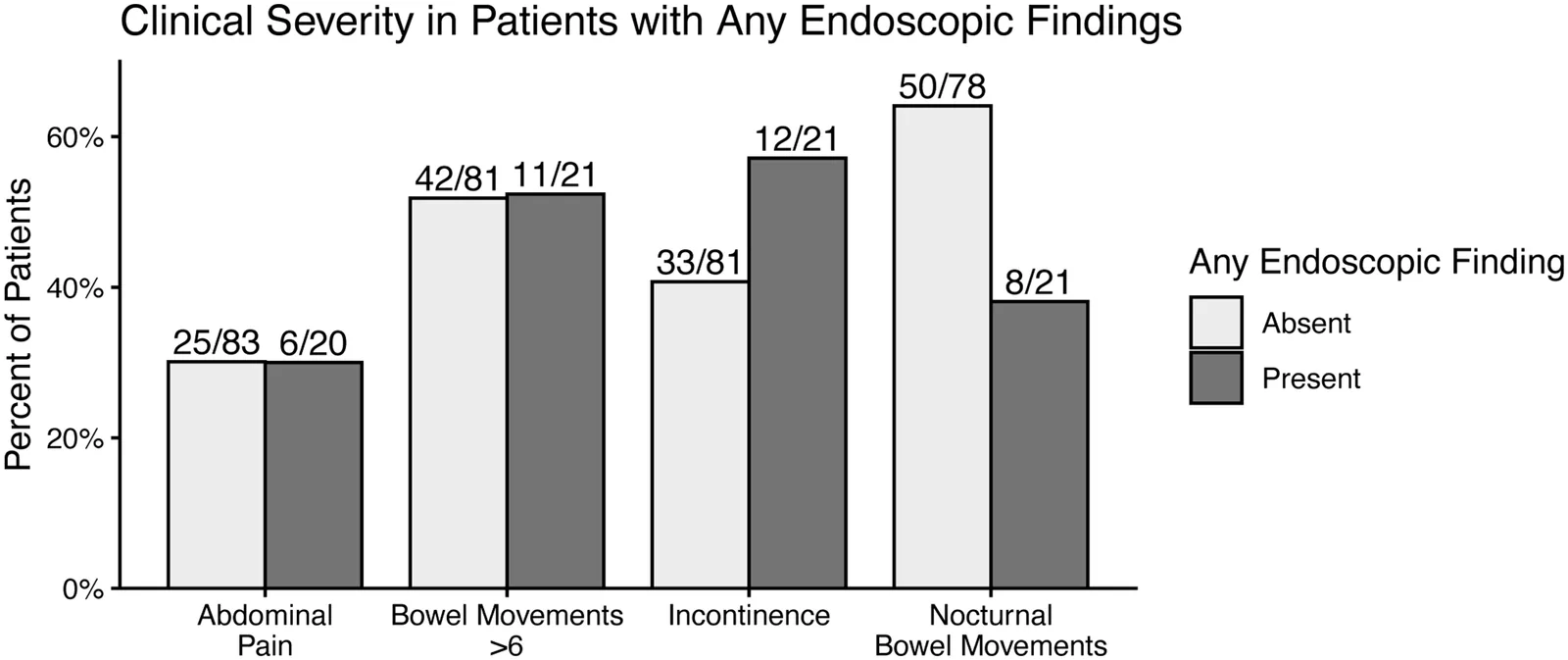
Endoscopic findings and severity of microscopic colitis symptoms. Prevalence of markers of MC severity (abdominal pain, greater than 6 bowel movements per day, incontinence, and nocturnal bowel movements) was compared between patients with and without endoscopic findings.

Of the 168 patients diagnosed with MC, 106 patients (63.1%) had data on symptoms collected at the time of their colonoscopy. Endoscopic findings were not significantly associated with symptoms of bowel movements greater than 6 per day, abdominal pain, incontinence, or nocturnal bowel movements (Figure 2).

## Discussion

Diagnostic colonoscopy with biopsies is a common tool used in the evaluation of chronic diarrhea and yields a specific diagnosis in 20% of cases, with the most common being MC in approximately 8% to 10% of cases.^19^ While biopsies are usually taken empirically to evaluate MC, endoscopic findings may help endoscopists to target biopsies, and with elevated suspicion, expedite appropriate therapy. Endoscopic findings have been reported in 10% to 30% of MC cases, with most findings being described as “subtle,” such as nonspecific mild erythema.^4^, ^5^, ^6^^,^^13^ A systematic review of published case reports identified that macroscopic findings seen in MC include mucosal lacerations/fractures, cicatricial lesions/scars, vascular changes (overcrowded or paucity of vasculature), and mosaic pattern/scalloping of mucosal folds, and pseudomembranes.^9^ We did not see any findings closely resembling pseudomembranes in our study despite case reports, so this was not included in our analysis. We found macroscopic findings in 24.4% of our patients with MC. Notably, we also saw endoscopic findings in 10.3% of patients without MC. This should not be surprising to most endoscopists, as subtle changes are frequently observed during routine screening colonoscopy or are often attributed as a normal variant in asymptomatic individuals. Thus, we felt that the use of a control group and consensus from a panel of gastroenterologists was crucial.

Among macroscopic findings, our panel found the least consensus with respect to vascular abnormalities. Whereas other macroscopic findings were more straightforward to identify, vascular variability is commonly observed during routine colonoscopy. In one large observational study, 108 of 607 patients with CC were noted to have macroscopic findings. However, 91 of 108 of those findings were vascular abnormalities.^2^ This study did not use a control group, so whether these subtle findings were seen more frequently in patients with MC was unclear. In our experience, there were no consistent vascular patterns seen in those MC patients with vascular abnormalities, from hypovascularity to vascular pruning to hypervascular findings, which likely explained our lack of consensus as well as inability to characterize a true vascular description to suggest a diagnosis of MC. However, we suspect that vascular findings on gross examination are dependent on chronic mucosal inflammation, as these findings are also common in patients with ulcerative or Crohn’s colitis.

Our panel most commonly agreed on the findings of nodular/mosaic pattern (Figure 1D) and scalloping (Figure 1E), perhaps given our experience with such findings in celiac disease. In our overall cohort, scalloping and abnormal vascularity were the only findings seen significantly more in MC compared to controls. Nodularity was twice as common in patients with MC than those without MC, but these differences did not reach statistical significance. Analogous to celiac disease, these findings are often seen in concert, as scalloping is used to describe mucosal folds and nodularity is often a similar finding but on flat mucosa. In our cohort, 15 of 16 patients with scalloping also had nodularity. One small study by Cimmino et al described mosaic pattern in MC and found an odds ratio of 19.4 in MC patients compared to controls; however, this finding was only seen in 4 patients in that study.^8^

While lacerations and scars were mostly obvious when seen, they were observed less frequently, 3 each in our MC population. Hemorrhagic lacerations have been linked to perforation after colonoscopy, especially in the setting of CC, and are postulated to result from the thicker collagen band causing increased stiffness of the colon.^9^, ^10^, ^11^ As these are rare adverse events, there were no perforations in our cohort, and no subjects reported clinically significant bleeding. Furthermore, other case reports have found similar hemorrhagic lacerations in normal colons without MC.^20^ For example, in the first reported series of “cat scratch colon” in 2007, 18 of 21 cases were actually in histologically normal colons, suggesting that these findings are not unique to MC.^21^

Analogous to endoscopic scoring systems in Crohn’s and ulcerative colitis, endoscopic/histologic predictors for more severe disease in MC are needed. Koulaouzidis et al identified that patients with MC with “plus symptoms” (weight loss, abdominal pain, bloating, fatigue, anemia, and elevated fecal calprotectin) were 3.2 times more likely to have mucosal erythema, but not other mucosal defects, than patients with diarrhea alone.^2^ Our study did not find an association between endoscopic findings and MC severity, particularly with the number of bowel movements, incontinence, and nocturnal bowel movements. Larger studies are needed to determine if either endoscopic or histologic findings can predict severity of symptoms at diagnosis or need for long-term budesonide therapy or escalation of treatment beyond budesonide.

Strengths of this study include the use of a control group with diarrhea, as subtle findings are not unique to MC. While the reviewers were blinded to patient diagnosis but not blinded to the purpose of this study, greater efforts to find abnormalities could have led to an overestimation of findings. However, we met afterward to come to a consensus on equivocal findings, and any overestimation would have been consistent between those with and without MC. Furthermore, patients with MC in our registry are well characterized, with detailed records of their endoscopy images, confirmation of diagnosis by histology, and clinical symptoms.

One weakness of the study was that endoscopic pictures were reviewed retrospectively, and thus endoscopic findings were dependent on a single endoscopist performing the procedure to either characterize these findings in their report or take representative digital photos for us to visualize. Thus, the true rate of endoscopic findings was likely underestimated with this approach, and prospective studies with multiple observers and/or video of endoscopy may increase reports of these findings. However, as our center has a focus on MC research, greater awareness of potential endoscopic findings increased the likelihood that representative photos were taken. Use of full video to better identify those with endoscopic findings is an area of future study. Lastly, while minor differences existed in the timing of collected clinical characteristics (eg, medication use) between patients with and without MC, recall bias was minimized by ensuring that all information was collected within 3 months of colonoscopy.

## Conclusion

To the best of our knowledge, this is the first study to compare all proposed endoscopic findings in MC to control patients with diarrhea. We found that abnormal vascularity, scalloping, or any macroscopic findings were more common in patients with MC than those without MC. However, when controlled for known risk factors of MC, this relationship was attenuated. Similar to other inflammatory bowel conditions, high-definition colonoscopy with currently integrated optical tools or chromoendoscopy may also improve our recognition of these findings.^22^ However, we feel that this analysis is sufficient to alert the readers to the subtle (or not so subtle) endoscopic findings in MC.
